# Construction and Curing Behavior of Underwater In Situ Repairing Coatings for Offshore Structures

**DOI:** 10.3390/polym16030306

**Published:** 2024-01-23

**Authors:** Yao Xu, Jiangbo Li, Yanxia Liu, Wei Wu

**Affiliations:** 1 State Key Laboratory of Simulation and Regulation of Water Cycle in River Basin, China Institute of Water Resources and Hydropower Research, Beijing 100038, China; xuyao@iwhr.com (Y.X.); liuyx@iwhr.com (Y.L.); 2 Key Laboratory of Engineering Materials of Ministry of Water Resources, Beijing 100038, China; 3 Engineering Center for Superlubricity, Jihua Laboratory, Foshan 528200, China; wuwei@jihualab.ac.cn

**Keywords:** underwater curing, adhesion, electrochemical impedance spectroscopy, coating

## Abstract

The development of polymeric materials for the repair and reinforcement of damaged sites in water has many practical applications, especially in ocean engineering. However, it is difficult to construct an anticorrosion coating in water. In addition, curing kinetics, which are the key to enhance the performance of coatings, seem to hardly be observed and regulated in an underwater condition. Herein, a novel underwater in situ repairing coating was prepared. Meanwhile, electrochemical impedance spectroscopy (EIS) was applied to observe its curing behavior underwater. Adhesion tests showed that the coatings cured underwater had good adhesion to different substrate surfaces and the ideal ratio of curing agent to epoxy resin was 0.6. Long-term anticorrosive tests demonstrated that the coatings had an excellent anti-corrosion performance. The viscosity changes in different curing stages were well reflected by frequency response characteristics from Bode and Nyquist curves by EIS. Two equivalent electrical circuits were selected to simulate the impedance date at the initial and final curing stage. A formula was put forward to evaluate the curing degree during the curing process. Finally, the effects of temperature and the ingredient ratio on the reaction rate and curing degree were also investigated here. This underwater in situ repairing coating may find applications in many offshore engineering structures in marine environments, and the EIS technique has attractive development and application prospects when observing the curing information of thermosetting resin systems under special circumstances.

## 1. Introduction

In recent decades, organic coatings have been extensively applied in ocean engineering structures to protect metal or concrete structures from corrosion [1,2]. However, surface coatings will inevitably be subjected to unexpected impact loadings during the service process, resulting in failure in protective performance [3,4]. If the damage is not treated in time, serious corrosion will occur on the exposed surface, especially for structures that are partially or completely immersed in seawater, such as offshore oil and gas platforms, offshore wind turbines, subsea pipelines, ocean-going vessels, and cross-sea bridges [5,6,7]. Given that the majority of offshore engineering buildings are located far from land, it is typically difficult to repair the damaged coatings using conventional remediation procedures [8,9]. Therefore, it is essential to develop novel coatings with an exceptional repair performance in marine environments.

Underwater in situ repairing coatings have recently attracted enormous research interest because they appear to be an ideal solution to the limitations of traditional underwater repair technology, including low accuracy, a multi-step process, the need for special apparatus, and subpar repair quality [10,11,12]. However, the hydration layer formed by water molecules on the substrate’s surface will prevent the molecular-level bridging between the coating and the substrate. As a result, it is difficult to form a strong underwater bonding [13,14]. Meanwhile, the contact area is macroscopically reduced by the trapped water at the interface. In addition, water will permeate into the coating during its solidification process in water, resulting in hydrolysis, swelling, and degradation of the coatings, which eventually leads to cohesive failure [15,16]. Several strategies have been proposed to develop underwater in situ repairing materials. The catechol-based acrylate polymer developed by Wilker et al. [17] was able to achieve a bonding strength of 3 MPa on aluminum underwater. Yang et al. [18] also prepared a novel underwater adhesive coating based on a layer-by-layer self-assembly technique. The coatings applied to the alloy surface exhibited an underwater adhesion strength of over 200 kPa for 1 day and more than 100 kPa for 60 days. Unfortunately, these aforementioned materials are difficult to use as anti-corrosion coatings due to their limited ability to resist the intrusion of both water and corrosive ions despite their excellent adhesive properties underwater.

Over the past decades, epoxy-based coatings, consisting of epoxy resin and a curing agent, have been successfully used in corrosion protection and emergency repair because of their remarkable chemical/corrosion resistance and mechanical performance [19,20,21] Recently, epoxy-based coatings have also been cured in wet and underwater environments by adjusting the hydrophilic and hydrophobic groups in the curing agent. Among all the curing agents, phenolic amines have drawn widespread attention. By adopting different amines and phenols, various curing agents with special structures and functions can be conveniently and highly efficiently created. For example, Li et al. developed a catechol-based Mannich base curing agent (CMB), which could be cured in a 4 °C underwater environment [22]. Zhou et al. also synthesized a variety of Mannich base curing agents and the maximum underwater bonding strength could reach 5.9 MPa [23].

Generally, the performances of resin-based composite materials are greatly dependent on their molecular structures [24,25,26], which are heavily controlled by the curing kinetics parameters, such as formulation, temperature, and curing time. Such curing kinetics parameters can provide a great deal of information about the ultimate structure and performance of the resin network, as well as the processing properties of the resin. Consequently, it is necessary to precisely observe and regulate the cure process in order to enhance the performance of the composites. However, traditional analytical methods such as differential scanning calorimetry (DSC), dielectric analysis (DEA), Raman spectroscopy and infrared spectroscopy (IR) appear to be less suitable for the underwater curing system. For instance, DSC results are susceptible to other events involving heat flow like water evaporation [27]. DEA requires a large number of prior experiments to ensure accuracy [28]. The results of Raman spectroscopy and infrared spectroscopy may also be disturbed by color, the refractive index, and other factors. In recent times, ultrasound elastography has been applied to investigate the process parameters and the physical properties of additive manufacturing products [29]. But it is not certain that it can be used to monitor the curing behavior of thermosetting resins. As a result, there is an urgent need to put forward a new analytical tool to offer real-time information on the curing reaction accurately and swiftly, especially for systems under special circumstances. More recently, Han et al. employed electrochemical impedance spectroscopy (EIS) to analyze the kinetic behavior of an epoxy resin [30,31]. It showed good concordance between the EIS and DSC results, indicating that EIS has great potential in monitoring the curing behavior of the thermosetting resin. However, the resin’s curing behavior was investigated in an air environment; it is unclear whether EIS can be used in other special curing conditions.

This currently reported study aimed to provide a new approach for the design of underwater in situ repairing coatings for offshore structures and was initiated to explore a new analytical method for the real-time monitoring of thermosetting resin systems under special circumstances. The underwater-curing epoxy-based coating was designed based on a phenolic amine curing agent synthesized by nonyl phenol (NP) and M-xylylenediamine (MXDA) through the Mannich reaction. The chemical structures of the curing agent were determined by Fourier-transform infrared spectroscopy (FTIR) and ^1^H-NMR spectroscopy. Meanwhile, a systematic study was carried out on the adhesion and anticorrosion properties of the coatings cured in various environments. Furthermore, electrochemical impedance spectroscopy (EIS) was utilized to observe the curing process in order to reveal the structure–property–processing relationship for the underwater curing systems. Two equivalent electrical circuits (EECs) were also simulated to provide information on the evolution of their viscosities, dielectric properties, and conductivities, during solidification. Finally, the influence of the temperature and the ratio of the curing agent to resin on the curing degree was researched to optimize the curing condition.

## 2. Experimental

### 2.1. Material

M-xylylenediamine (MXDA, 99%) and nonyl phenol (NP, ≥98%) were purchased from Shanghai Macklin Biochemical Co., Ltd. (Shanghai, China). Formaldehyde (37 wt% sol in water) and sodium chloride were obtained from Tianjin Damao Chemical Reagent Factory (Tianjing, China). The NPEF-170 epoxy resin (epoxy value, 0.57–0.59 mol/100 g) was supplied by Nan Ya Plastics Industrial Co., Ltd. (Taipei, China). The wetting dispersing agent (Solsperse 19000), defoamer (KMT-2233), organic bentonite (TY-168B), rutile titanium dioxide (DHR-966), ultrafine quartz powder (1250 mesh), talcum powder (1200 mesh) and precipitated barium sulphate were purchased from Shanghai King Chemical Co., Ltd. (Shanghai, China). The silane coupling agent (KH-560, 97%) was purchased from Jiangxi Chenguang New Materials Co., Ltd. (Jiujiang, China). All commercial chemicals and solvents were used as received without further purification.

### 2.2. Synthesis of Mannich-Modified m-Xylylenediamine (m-MXDA)

To synthesize the curing agent, NP (26.44 g, 1.2 mol) and MXDA (15.14 g, 1.4 mol) were mixed and added to a four-necked flask. The mixture was then heated to 60 °C and maintained at this temperature for 1 h. Next, a 37 wt% formaldehyde solution (6.38 g, CH_2_O: 0.8 mol) was added dropwise to the flask at 80 °C. The temperature was then increased to 100 °C, and the mixture was refluxed for 4 h. After the refluxing process, the water content was reduced by vacuum distillation for 2 h at temperatures up to 120 °C. The resulting brown-yellow liquid obtained was used directly as the curing agent in the subsequent experiments.

### 2.3. Preparation of the In Situ Underwater Repairing Coating

The coating consisted of components A and B, where A was composed of epoxy, diluent, pigments, fillers, and additives, while B was made up of the m-MXDA above. Component A was prepared as follows: bisphenol F epoxy resin (50 wt%), reactive diluent (4 wt%), wetting dispersing agent (0.5 wt%), defoamer (0.5 wt%) and organic bentonite (1 wt%) were pre-mixed in a high-speed agitator for 45 min at a speed of 800 r/min. Subsequently, rutile titanium dioxide (10 wt%), ultrafine quartz powder (13 wt%), talc powder (10 wt%), precipitated barium sulfate (10 wt%) and KH-560 (1 wt%) were successively added, and the mixture was thoroughly dispersed at a speed of 1500 r/min for 2 h. The homogenized epoxy composite was then ground using a three-roll grinder. Components A and B were combined in a stoichiometric ratio, and the coating was stirred thoroughly for 15 min before application. The coating was denoted as A-Bx-y, where x represents the weight ratio of component B to bisphenol F epoxy resin, and y represents the curing temperature.

The above coating was painted on the surface of the substrate using wire rod brushings. For adhesion and long-term immersion tests, the thickness of the dry film was kept at 80 ± 5 μm. Carbon steel was chosen as the substrate where the sandblasted steel plate was sand-blasted to meet the requirements of Swedish specification SA 2.5, while the low-surface-treatment steel was polished using 320# abrasive paper to remove all the surface oxides. For dynamical mechanical tests, the coatings were prepared on a polyethylene film and peeled off after curing for 7 days at 25 °C. Then, the thin films were cut into 10 × 5 mm^2^ for testing. Deionized water and 3.5 wt% NaCl solution were selected to make up the underwater construction environment.

### 2.4. Characterization

#### 2.4.1. Fourier-Transform Infrared Spectroscopy (FTIR)

FTIR spectra were obtained by a Nexus 470 IR spectrometer (Thermo Nicolet, Waltham, MA, USA). A KBr disk was used and the wavenumber range was 400–4000 cm^−1^.

#### 2.4.2. Nuclear Magnetic Resonance Spectroscopy

^1^H-NMR spectra were conducted on a Bruker AVANCE III 500 instrument using CDCl_3_ as solvent.

#### 2.4.3. Pull-Off Tests

The adhesion between the coating film and the substrate (mortar or carbon steel) was carried out using the PosiTest AT-A pull-off adhesion tester (DeFelsko, Ogdensburg, NY, USA) based on ASTM D7234 [32]. The diameter of the measurement area was 20 mm and the pulling speed was 0.2 MPa/s. Each sample was measured 5 times to obtain an average value.

#### 2.4.4. Dynamical Mechanical Analysis (DMA)

DMA was performed on a DMA Q800 instrument (TA Instruments, New Castle, DE, USA). Samples were tested from −75 °C to 200 °C. The heating rate was 5 °C/min and the frequency was 1 Hz. Nitrogen was used as the test atmosphere.

#### 2.4.5. Electrochemical Impedance Spectroscopy (EIS) Measurements

All EIS measurements were carried out at an electrochemical workstation (GMARY Reference-620, Warminster, PA, USA) with a three-electrode system. A saturated Ag/AgCl electrode and platinum sheet were chosen as the reference electrode and counter electrode, respectively. The EIS measurements were tested in a 3.5 wt% NaCl solution in the range of 10^−2^ Hz to 10^5^ Hz, with an amplitude sinusoidal voltage of 10 mV. For anti-corrosive tests, carbon steel was chosen as the working electrode and the thickness of dry film was 80 ± 5 μm. For in situ underwater curing behavior tests, graphite was chosen as the working electrode to avoid the influence of corrosion, and the thickness of the uncured sample was 100 ± 5 μm. In situ EIS tests were carried at an interval of 15 min using Sequence Wizard script. A temperature control system (JULABO 200F, Seelbach, Germany) was used to keep the temperature constant during the tests. Finally, the EIS data were analyzed and fitted into equivalent electrical circuits (EEC) by Z_SimpleWin_ software 3.30.

## 3. Results and Discussion

### 3.1. Synthesis and Characterization of Mannich-Modified m-Xylylenediamine (m-MXDA)

**Figure 1:**
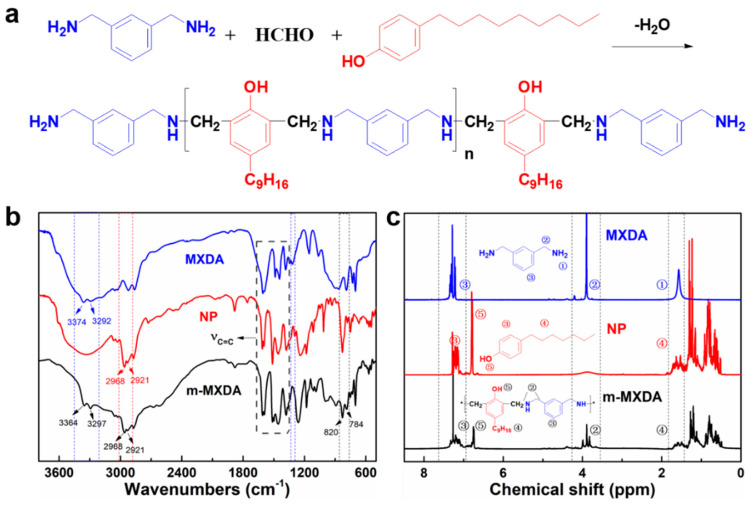
(**a**) The route for synthesis of nonylphenol modified MXDA (m−MXDA) through the Mannich reaction; (**b**) FTIR spectra of MXDA, NP and m−MXDA; and (**c**) ^1^H-NMR spectra of MXDA, NP and m−MXDA.

Figure 1a depicts the diagram of the Mannich reaction between NP, formaldehyde, and MXDA to prepare modified MXDA (named as m-MXDA). Figure 1b shows the FTIR spectra of m-MXDA, MXDA, and NP. The peaks at 1589 cm^−1^, 1460 cm^−1^, and 1424 cm^−1^ belong to the stretching vibrational absorption of -C=C in the skeleton, indicating the three chemicals all have a phenyl structure. There are two similar absorption peaks near 3374 cm^−1^ and 3202 cm^−1^ in the spectrum of MXDA and m-MXDA, which are derived from the N-H stretching vibrations of primary and secondary amines. Compared to MXDA, m-MXDA exhibits two new absorption peaks at 781 cm^−1^ and 820 cm^−1^ in the fingerprint region, which are the bending vibration peaks of -C-H from ortho-substituted and meta-substituted benzene rings. Moreover, there are also antisymmetric and symmetric stretching vibration peaks at 2968 cm^−1^ and 2921 cm^−1^, respectively, which are derived from the C_9_ aliphatic chain in nonylphenol. In Figure 1c, the peak of -NH_2_ (1.51 ppm) is attenuated in the ^1^HNMR spectrum of m-MXDA, while the peak of phenolic hydroxyl (6.75 ppm) exists. The peak of -CH_2_- is cleaved due to the formation of new methylene groups. All of these results indicate that the modified m-xylylenediamine was successfully prepared through the Mannich reaction.

### 3.2. Adhesion and Properties of the Underwater In Situ Repairing Coatings

**Figure 2:**
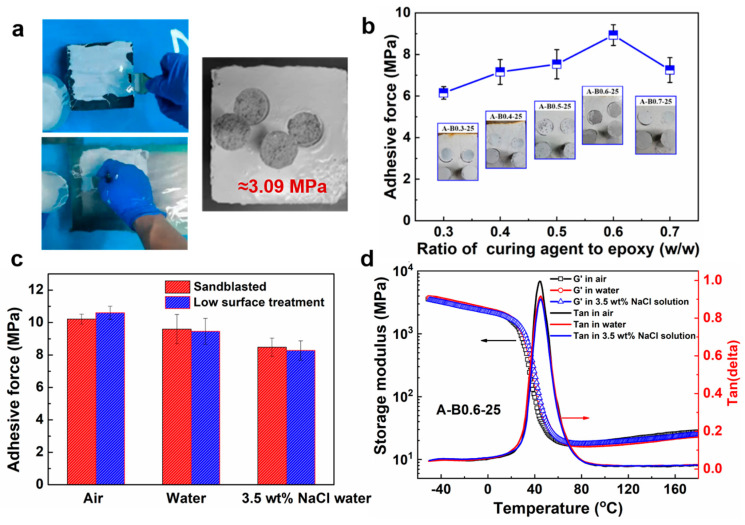
(**a**) Underwater application of the coating on concrete and steel structure and adhesion test of the coating on concrete structure; (**b**) effects of ratio of curing agent to epoxy resin on underwater adhesion force on steel structure; (**c**) effects of pretreatment and curing environment on adhesion force; and (**d**) DMA measurements of sample A-B0.6-25 cured in different environments.

As can be seen in Figure 2a, the prepared coating can be applied to both the surface of the concrete and steel structures in the underwater condition without dispersing or solvent escaping. The average bonding strength value on the surface of the concrete was approximately 3.09 MPa and fractures occurred in the substrate, indicating that the coating has excellent adhesion to the concrete structure. Figure 2b illustrates the bonding strength on the steel structure varying with different ratios of curing agent to epoxy resin. When the curing agent ratio increased from 0.3 to 0.6, the adhesion increased gradually and the fracture pattern changed from cohesive failure within the coating to interface failure between the coating and substrate. The adhesion weakened and the fracture pattern reverted to cohesive failure in the coating when the ratio was further increased. As is well known, increasing the ratio of curing agent to epoxy resin is beneficial for the formation of a cross-linking network, which results in the improved cohesive strength of the coating [33,34]. Nevertheless, in the case of the brittle epoxy system with low chain flexibility, a high concentration of the curing agent can also result in excessive cross-linking density, leading to counter-productive results [35]. Therefore, a maximum adhesion of about 8.3 MPa can be obtained at an optimized ratio of 0.6.

In Figure 2c, no significant difference in adhesion is observed between the substrates treated by sandblasting and those treated by simple polishing, regardless of whether the coating was prepared in air, water, or 3.5 wt% NaCl solution. This feature is crucial because the structure cannot be a high-profile surface prepared in most underwater in situ repairing cases [36]. Nevertheless, the adhesive force of the coating prepared in 3.5 wt% NaCl solution was significantly lower compared to that in water or air. The decrease in adhesion could be attributed to the unavoidable corrosion during the preparation process. As shown in Figure 2d, the storage modulus of the three coatings, as determined from DMA tests, almost overlapped during the entire temperature range. In addition, the maximum temperature of the loss factor, which represents the glass transition temperature, for all three coatings, was nearly identical, demonstrating that the solidification environment has little effect on the mechanical properties of the coatings.

### 3.3. Anticorrosive Property of the Underwater In Situ Repairing Coatings

**Figure 3:**
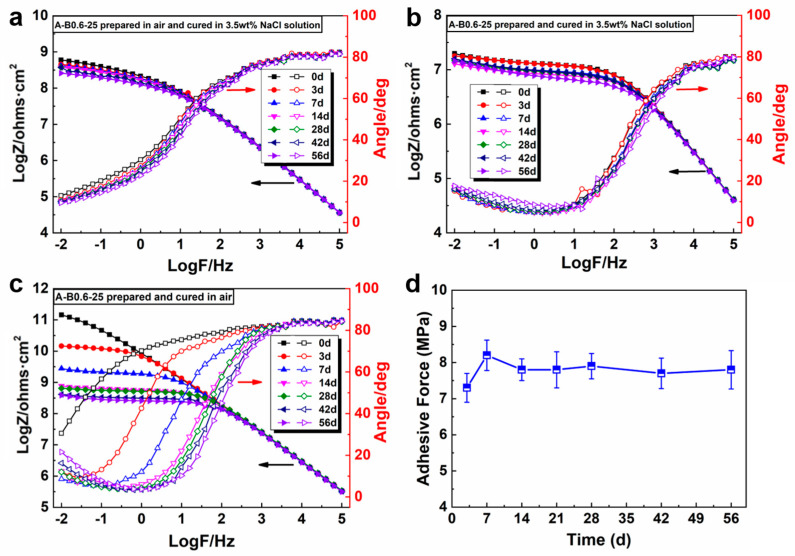
Bode plots of sample A-B0.6-25 prepared and cured in different environments during 3.5 wt% NaCl solution immersion: (**a**) both prepared and cured in 3.5 wt% NaCl solution, (**b**) prepared in air and cured in 3.5 wt% NaCl solution, and (**c**) both prepared and cured in air; (**d**) evolution of adhesion for sample A-B0.6-25 prepared and cured in 3.5 wt% NaCl solution during long-term brine soaking.

EIS measurements were performed to track the deterioration of the anti-corrosion properties of the coatings in 3.5 wt% NaCl solution, where A-B0.6-25 was taken as a representative example. Figure 3 illustrates the Bode plots of A-B0.6-25 prepared and cured in different environments during immersion. In terms of the coatings cured in 3.5 wt% NaCl solution (Figure 3a,b), there is an obvious plateau in the initial stage of the Bode plots and two time constants can be observed in the phase diagrams, revealing that corrosion had occurred in the coating/steel interface [37]. The corrosion came from the flash rust due to contact with water during the preparation process. A similar phenomenon can also be observed on the sample prepared in air and cured in saline solution (Figure 3b). With regard to coating prepared and cured in air (Figure 3c), the impedance modulus spectrum at the initial stage possessed a high resistance of about 10^11^ Ω·cm^2^ at a low-frequency range, exhibiting approximately a quasi-capacitive characteristic. Meanwhile, a higher phase angle at a high frequency and a wider frequency region with an 80 phase angle was observed. After 14 d of exposure, the impedance modulus decreased rapidly to a level of 10^8^ Ω·cm^2^ with time, and the phase angle at the lower-frequency region shifted to higher frequencies. The second time constant appeared after 3d. This was due to micron-sized defects in the coating which provided the pathways for water and ions, leading to the declined barrier performance [38]. However, for all coatings, the shrinkage rate of the Bode plots was restrained to a certain degree and the impedance of all the coatings at the low-frequency range was above 10^7^ Ω·cm^2^ during the immersion time of 56 days, implying that the coatings still had excellent corrosion resistance ability. Moreover, the adhesive force of the coatings prepared and cured in 3.5 wt% NaCl solution remained at about 7.8 MPa (Figure 3d) without a significant reduction during the whole soaking time, proving that the underwater in situ repairing coating can be used for long-term anticorrosion in marine environments.

### 3.4. Curing Behavior of the Underwater In Situ Repairing Coatings in 3.5 wt% NaCl Solution

#### 3.4.1. Theoretical Basis of Measuring Curing Behavior of Thermosetting Resin Using Electrochemical Impedance Spectroscopy (EIS)

Generally, the dielectric loss of a thermosetting resin mainly comes from the motion of ions and the orientation of dipoles [39,40]. Before applying an electric field, the ions and dipoles initially undergo a random state in the resin. When subjected to an electric field, the ions start to move towards an electrode of opposite polarity while the dipoles try to align with the electric field. Therefore, the dielectric loss can be expressed by the following equations:

(1) $$\varepsilon^{\prime \prime }=\varepsilon_{d}^{\prime \prime }+\varepsilon_{i}^{\prime \prime }$$

(2) $$\varepsilon_{i}^{\prime \prime }=\frac{\sigma }{\omega \varepsilon_{0}}=\frac{\sigma }{2\pi f\varepsilon_{0}}$$

where *ε*″ is the dielectric loss factor of the resin; *ε_d_*″ is the dipolar polarization contribution; *ε_i_*″ is the ionic polarization contribution; *σ* is the electrical conductivity of the thermosetting resin; *ε*_0_ is the dielectric constant of the vacuum and *f* is the applied frequency.

As the reaction proceeds, the thermosetting resin undergoes a gradual transition from a liquid state to a solid state. Therefore, the migration of ions and the rotation of dipoles are increasingly restricted with the reaction going on. Since ion conductivity comes from ion mobility, which is highly dependent on the viscosity of the material, it can well reflect the viscosity change during the curing process [28]. In addition, the dielectric loss factor at a very low frequency is predominantly dependent on ion migration while the contribution of dipole orientation can be ignored [41]. Thus, the degree of cure can be calculated by measuring the change in the dielectric loss factor and the following formula is proposed [30]:

(3) $$\left(t\right)=\frac{\log\left(\varepsilon_{0}^{\prime \prime }\right)-log(\varepsilon_{t}^{\prime \prime })}{\log\left(e_{0}^{\prime \prime }\right)-log(e_{\infty }^{\prime \prime })}$$

where *ε*_0_″ is the dielectric loss factor of the thermosetting resin before the curing; *ε_t_*″ is the dielectric loss factor of the thermosetting resin cured after time *t*; *ε*_∞_″ is the dielectric loss factor of the thermosetting resin after being fully cured; and *α(t)* refers to the curing degree of the thermosetting resin at time *t*.

In the AC electrical field, the hindrance to AC can be represented as the impedance modulus. Since there is a negative correlation between electrical conductivity and impedance modulus, Equation (1) can be converted into Equation (4) when the frequency is low.

(4) $$\varepsilon^{\prime \prime }=\frac{\sigma }{2\pi f\varepsilon_{0}}=AZ^{-1}$$

where *Z* is the impedance modulus; *A* is the specific and temperature-dependent constant of thermosetting resin.

Thus, the curing degree can be measured using the impedance modulus as variable by substituting Equation (4) into Equation (3):

(5) $$\alpha \left(t\right)=\frac{\log\left(Z_{t}\right)-\log\left(Z_{0}\right)}{\log\left(Z_{\infty }\right)-log(Z_{0})}$$

where *Z_t_* is the impedance modulus of resin cured after time *t*; *Z*_0_ is the impedance modulus of resin at the beginning; and *Z_∞_* is the impedance modulus at infinity.

#### 3.4.2. EIS Monitoring of the Coatings Cured in 3.5 wt% NaCl Solution

**Figure 4:**
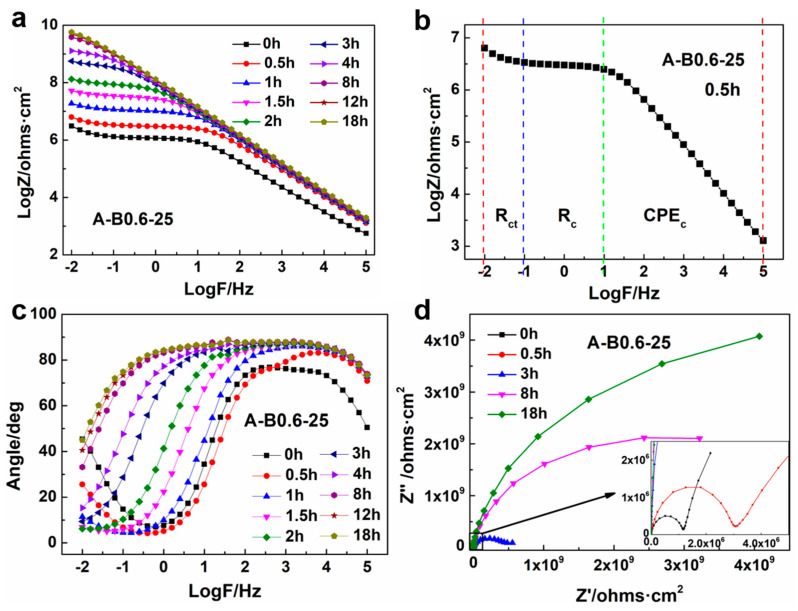
(**a**) Bode plots of sample A-B0.6-25 at different curing times. (**b**) Frequency response characteristics of sample A-B0.6-25 at initial curing stage (0.5 h). (**c**) Phase diagrams of sample A-B0.6-25 at different curing times. (**d**) Nyquist plots of sample A-B0.6-25 at different curing times.

EIS measurements were conducted to monitor the isothermal curing process of the uncured samples under constant temperature conditions in 3.5 wt% NaCl solution. Graphite was chosen as the working electrode for the purpose of eliminating the interference of corrosion. The measured impedance data are presented in the form of Bode plots to describe the frequency dependence of impedance, as seen in Figure 4a. It is noted that the impedance increased with an increasing curing time and finally remained almost constant over the entire test frequency range. The system’s fluidity diminished and its viscosity rose as the reaction went on. Accordingly, the mobility of the charged particles got hindered, leading to reduced conductivity and increased impedance. Finally, the impedance remained stable once the system was fully cured and a complete three-dimensional network structure was formed.

It is also evident that the Bode plots exhibit different shapes at different curing stages. In the initial stage of curing (0.5 h, Figure 4b), the Bode plot comprises three regions. At low frequencies such as 10^−2^–10^1^ Hz, the impedance increased as the frequency decreased, and it was mainly controlled by ion diffusion. Within the intermediate frequency region of 10^−1^–10^1^ Hz, the impedance remained almost unchanged since it mainly depended on the resistance of the system, which is closely associated with the reaction kinetics. During high frequencies such as 10^1^–10^5^ Hz, the impedance continued to increase as the frequency decreased, and the Bode plot is analogous to a straight line, with a slope of −1. When the reaction reached a medium curing stage (≥2 h), only two parts exist in the Bode plot, where the impedance grew within the high-frequency range and stayed the same in the low-frequency range. From Figure 4a, it can be seen that the point of inflection shifted from high frequency to low frequency as the reaction proceeded. Finally, the Bode plot is approximate to a straight line with a slope of −1 in the whole-frequency range at the later curing stage (such as 18 h).

As the curing progressed, the phase angle close to 90° gradually shifted towards lower frequencies, indicating that the electrochemical process slowed down and a three-dimensional network was formed. At the same time, the phase angle approached 90° over a wide frequency region in the final stage of curing, indicating that a complete and dense coating had been formed which was close to an insulating medium and can be approximated as a capacitive element in the electrode system [31]. The Nyquist plots shown in Figure 4d clearly demonstrate the electrochemical behavior as well as the cross-linking degree of the system. In the initial curing stage, two capacitive arcs can be observed. The one located in the high-frequency region is related to the capacitance characteristics of the resin system while the other in the low-frequency region is relevant to the charge transfer resistance of the system. In the late curing stage, only one arc exists in the Nyquist plot, which reflects the capacitance characteristics of the resin system. As the curing time went on, the mobility of ions was restricted and the charge transfer resistance was increased as a result of the improved crosslinking of the system. Thus, the diameter of the capacitance arc was significantly increased and the Nyquist plots move closer to the Y-axis over time, demonstrating that the electrochemical setup mainly exhibited capacitive characteristics and the cured resin film on the electrodes can be regarded as an insulating separator in the final stage of the reaction.

#### 3.4.3. Equivalent Circuit Simulation of Coatings with Different Underwater Curing Times

**Figure 5:**
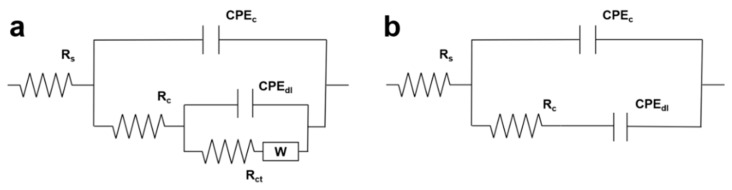
EEC models selected to simulate impedance data of sample A-B0.6-25 being cured at (**a**) initial stage and (**b**) end stage.

The equivalent electrical circuits were employed to further analyze the AC impedance results and evaluate the behavior of the coating. Two EEC models were selected to simulate the impedance date at the initial curing time and the final curing stage, as illustrated in Figure 5. All capacitance elements were replaced by constant phase elements (CPE) to correct the non-pure capacitance originating from the surface inhomogeneity and roughness [42,43]. The electrochemical elements *R_s_*, *CPE_c_*, *R_c_*, *CPE_dl_*, *R_ct_*, and *W* in the equivalent circuit models represent solution resistance, resin capacitance, resin resistance, coating/electrode interface capacitance, charge transfer resistance and diffusion impedance, respectively. *CPE_c_* and *CPE_dl_* are related to the dielectric properties of the coating during curing. *R_ct_* is associated with the mobility of ions, which is controlled by the viscosity of the system. *R_c_* characterizes the conductivity and insulation of the system. *W* is the Warburg impedance caused by micropore diffusion due to the lack of density of the coating in the early curing stage.

**Figure 6:**
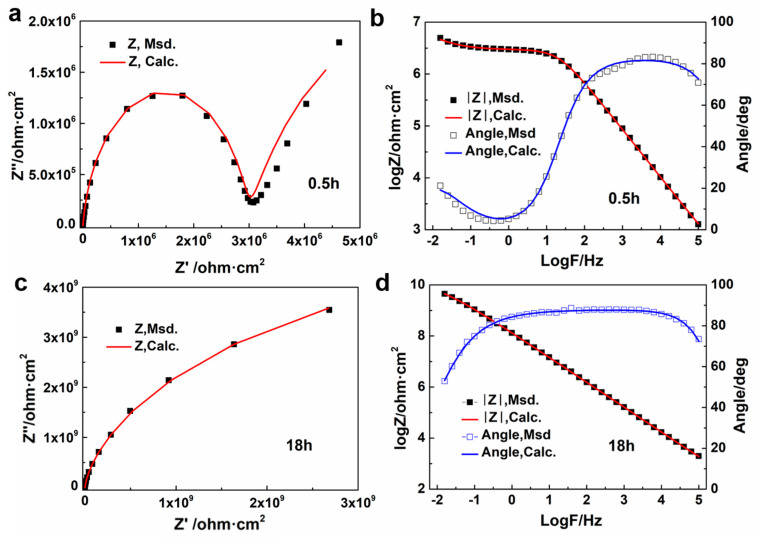
EEC simulation results for Nyquist plots (**a**,**c**), impedance modulus and phase angle (**b**,**d**) of sample A-B0.6-25 at 0.5 h (**a**,**b**) and 18 h (**c**,**d**).

All the fitting data are shown in Table 1. As the curing reaction progressed, the viscosity of the system rose and the conductivity of the coating decreased due to the restriction of the mobility of ions and charge transfer, which leads to the increasing of *R_c_* and *R_ct_*. In Figure 6, there is a strong consistency between the experimental data and the fitting data, indicating that the chosen equivalent circuit model has high accuracy and reliability.

#### 3.4.4. Curing Degree of Different Coating Systems

**Figure 7:**
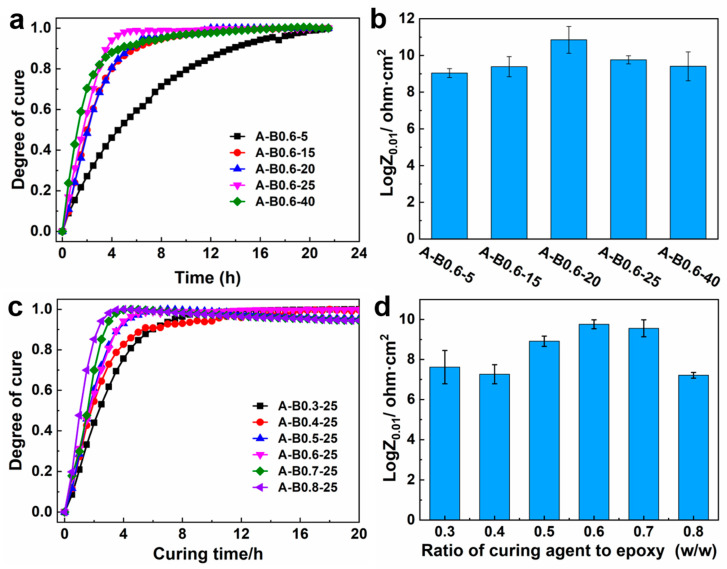
(**a**) Curing degree of A-B0.6 cured in different temperatures as function of cure time. (**b**) The impedance value at 10^−2^ frequency of A-B0.6 cured in different temperatures. (**c**) Curing degree of the coatings varying in different ratios of curing agent to epoxy resin as a function of cure time. (**d**) The impedance value at 10^−2^ frequency of coatings varying in different ratios of curing agent.

The Bode plots of the coatings cured at different temperatures and various ratios are shown in Figures S1 and S2. The curing degree was calculated using Formula (5) and plotted as a function of curing time in Figure 7a. It is evident that the degree of curing was accelerated with the increase in temperature (5 to 25 °C), which agrees with the exothermic curing reaction characteristics of epoxy resin. However, the curing degree at a later stage was lower at a higher temperature (such as 40 °C) than it was at 25 °C. Although a higher temperature expedited the reaction, the viscosity sharply rose, which limited the further reaction of epoxy and amino residues in the system. As a result, the reaction rate became slower in the later stage. It is likely that the optimal curing temperature was 20 °C, as shown in Figure 7b, where the maximum logZ_0.01_ could be achieved. All of the coatings had impedance values between 10^9^ and 10^10^ at 10^−2^ frequency, suggesting that they can all form a full network structure.

It can also be seen in Figure 7c that the reaction rate of the system accelerates with the increase in curing agent concentration. The optimal curing agent concentration was 0.6 where the obtained logZ_0.01_ was highest. There was still an approximate platform of Bode plots in the low-frequency region even the reaction in A-B0.3-25 and A-B0.8-25 reached equilibrium (Figure S2). Meanwhile, the impedance value at 10^−2^ frequency was within 10^7^~10^8^, which was one order of magnitude smaller than that in A-B0.6-25. This suggests that the densification of the coating network structure will be impacted by either an excessive or insufficient amount of curing agent. In general, all the systems reached curing equilibrium within 8~12 h, demonstrating that the high reactivity of the Mannich-modified amines facilitates the quick formation of coatings in the underwater environment.

The aforementioned findings indicated that EIS is an effective and alternative approach to monitor the curing processes of thermosetting resin systems, providing curing kinetics parameters which can help guide the formulation optimization, production and application. Compared with existing technologies, EIS seems to have a wider range of applications, especially for systems cured in special conditions, because it can avoid some interferences of the outside. However, more research is still needed to confirm its accuracy and reliability. Future studies will focus on comparing these results with other technologies. The research findings will be reported in due course.

## 4. Conclusions

In this study, we have demonstrated an underwater in situ repairing coating with excellent anticorrosion properties. Meanwhile, EIS was used to monitor the curing processes of coatings underwater. The main results were as follows:

(1) The prepared coatings had prominent adherence adhesion to the substrate whether in air, water, or 3.5 wt% NaCl solution. DMA analysis indicated that the mechanical properties of the coatings were not significantly impacted by the solidification environment. In addition, EIS results showed that the coatings exhibited excellent anticorrosion properties and could be applied to marine environments for long-term corrosion prevention.
(2) Viscosity is a great important physical parameter during the curing period. Since impedance modules are attributed to ion mobility, which is related to the viscosity of the medium, electrochemical impedance spectroscopy (EIS) can be used to measure the curing behavior of a thermosetting resin. A formula, using the impedance modules as the primary variable, was put forward to evaluate the curing degree during the curing process.
(3) The viscosity changes were well reflected by frequency response characteristics from Bode and Nyquist curves by EIS in different curing stages. Two EEC models were chosen to simulate the impedance date at the initial and final curing stage.
(4) The curing degree increased obviously with increasing temperature. However, the dramatically increased viscosity would limit the further reaction of epoxy and amino residues in the system at a higher temperature. The ingredient ratio also had a significant influence on the reaction rate and the system. The optimal temperature and ratio were 20 °C and 0.6, respectively.

## Figures and Tables

**Table 1:** Electrochemical parameters extracted from EIS data sample A-B0.6-25 at different curing time.

Parameter	0.5 h	2 h	8 h	18 h
*R_s_* (Ω·cm^−2^)	224.5	351	412.2	508.4
*CPE_c_* (S·cm^−2^·s^n^) *	3.83 × 10^−9^	1.79 × 10^−9^	6.14 × 10^−10^	2.35 × 10^−10^
*R_c_* (Ω·cm^−2^)	22.98 × 10^6^	3.02 × 10^7^	1.44 × 10^9^	1.43 × 10^10^
*CPE_dl_* (S·cm^−2^·s^n^) *	2.07 × 10^−6^	8.88 × 10^−9^	1.44 × 10^−9^	1.20 × 10^−9^
*R_ct_* (Ω·cm^−2^)	6.09 × 10^6^	1.16 × 10^8^	--	--

*: n represent the deviation between constant phase element (*CPE*) and capacitance element (*C*), which can also be regarded as the degree of surface inhomogeneity and roughness. The value ranges from 0 to 1.

## Data Availability

Data are contained within the article and Supplementary Materials.

## Supplementary Materials

The following supporting information can be downloaded at: https://www.mdpi.com/article/10.3390/polym16030306/s1, Figure S1: Bode plots of the coatings cured in different temperatures during the curing process; Figure S2: Bode plots of the coatings varying in different ratios during the curing process.
