# Supplementary material for: Construction and Curing Behavior of Underwater In Situ Repairing Coatings for Offshore Structures
Source: Polymers (Basel). 2024 Jan 23;16(3):306. doi: 10.3390/polym16030306 (PMC10857492; doi:10.3390/polym16030306)
Supplement: Supplementary file 1 [file polymers-16-00306-s001.zip › polymers-2801818-supplementary.pdf]

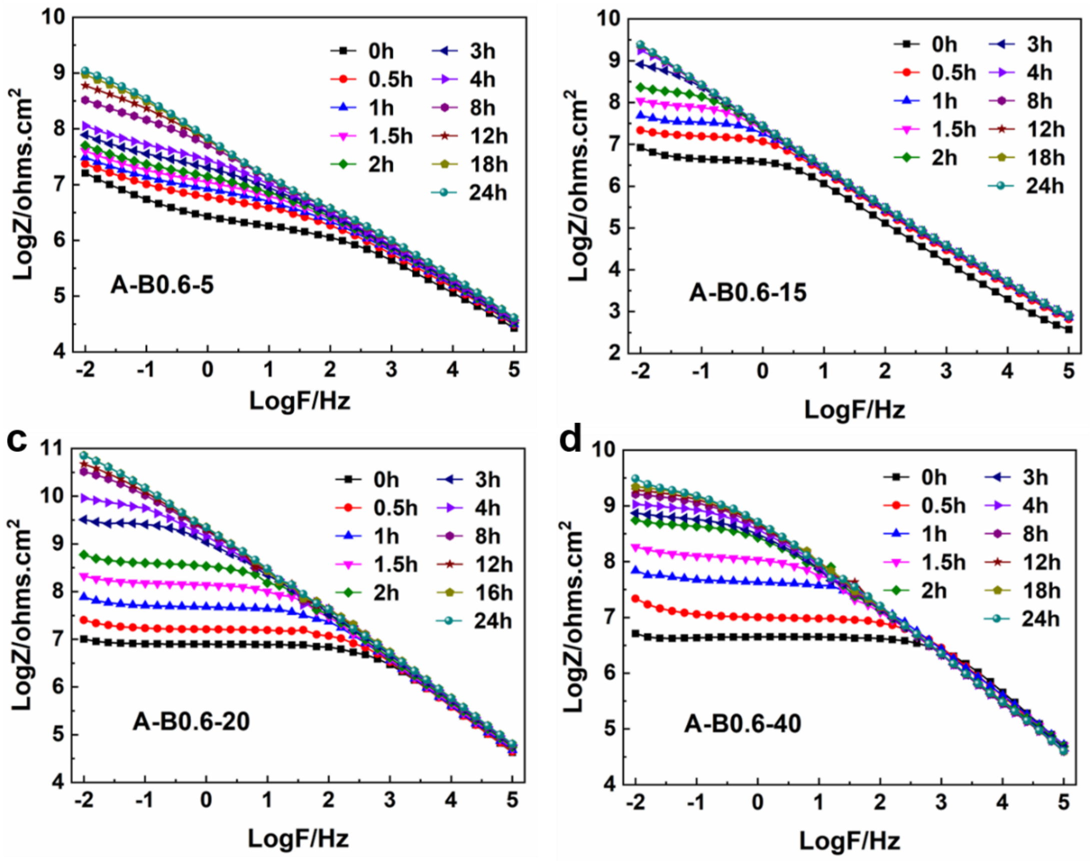

Figure S1. Bode plots of the coatings cured in different temperatures during the curing process.

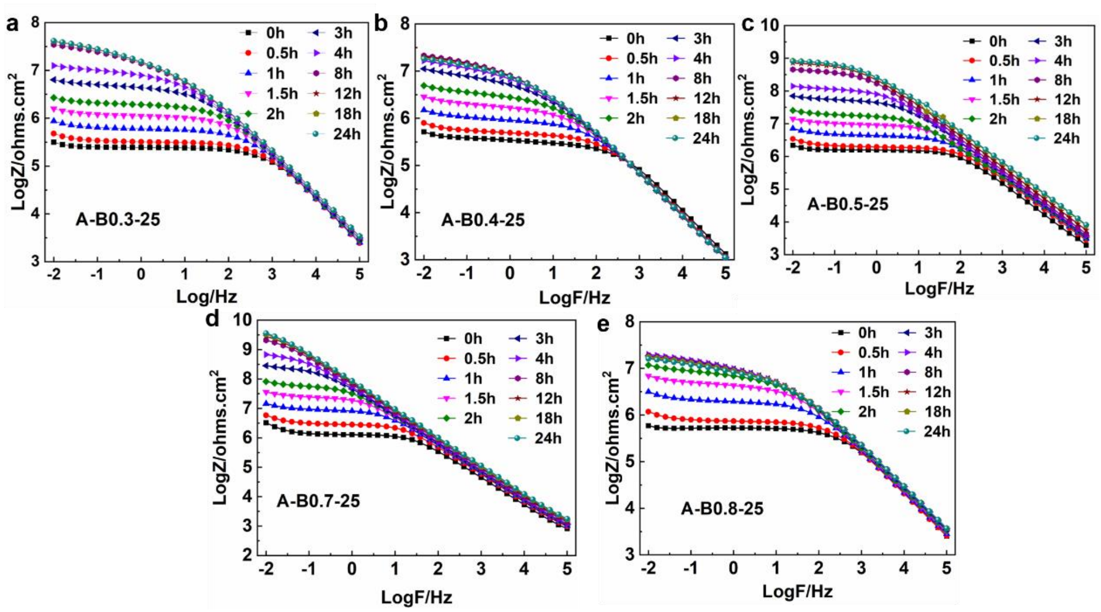

Figure S2. Bode plots of the coatings varying in different ratios during the curing process.
